# Umbilical cord androgens and estrogens in relation to verbal and nonverbal abilities at age 10 in the general population

**DOI:** 10.1371/journal.pone.0173493

**Published:** 2017-03-09

**Authors:** Esha S. L. Jamnadass, Jeffrey A. Keelan, Suzanna N. Russell-Smith, Martha Hickey, Murray T. Maybery, Andrew J. O. Whitehouse

**Affiliations:** 1 School of Psychology, University of Western Australia, Perth, Western Australia, Australia; 2 Telethon Kids Institute, University of Western Australia, Perth, Western Australia, Australia; 3 School of Women’s and Infant’s Health, University of Western Australia, Perth, Western Australia, Australia; 4 Department of Obstetrics and Gynaecology, University of Melbourne and the Royal Women’s Hospital, Melbourne, Victoria, Australia; Western University of Health Sciences, UNITED STATES

## Abstract

Sex differences in verbal and nonverbal abilities are a contentious area of research. Prenatal steroids have been shown to have masculinizing effects on the brain that may affect the development of nonverbal and verbal abilities in later life. The current study examined a wide range of biologically active sex steroids (both androgens and estrogens) in umbilical cord blood at birth in a large pregnancy cohort in relation to performance on nonverbal (Raven’s Coloured Progressive Matrices) and verbal (Clinical Evaluation of Language Fundamentals-3 and the Peabody Picture Vocabulary Test-III) measures at age 10 years. Overall, Androgen and Estrogen composites in cord blood were not found to be predictive of performance on verbal and nonverbal measures at age 10. These data suggest that late gestation sex steroids do not exert a major effect on nonverbal and verbal abilities in middle childhood.

## Background

Sex differences in cognition have been a long standing subject of investigation [1–4]. One aspect of interest is that of sex differentiated performance in nonverbal versus verbal domains. Males have been found to outperform females in nonverbal tasks such as spatial perception, spatial visualization [5] and mental rotation tasks [6–9]. Conversely, females have been shown to outperform males in the domain of verbal abilities [1] in tasks such as verbal fluency [10] and vocabulary [11]. However, there remains significant variation in the size and presence of effects that is dependent on the task and sample involved [5, 12, 13].

Possible causes for these cognitive sex differences have seen extensive research attention [1, 14]. There has been growing interest in the impact of biological influences, such as sex steroids, on the developing brain during prenatal life [15, 16]. Sex steroids are hypothesized to exert an ‘organisational effect’ on the fetal brain that has long lasting effects on cognition [17, 18]. Examining prenatal sex steroids in relation to cognition not only enhances our understanding of the mechanisms underlying typical brain development, but may also expand our knowledge of prenatal factors affecting atypical neurodevelopmental conditions such as Autism Spectrum Disorder [19–21].

Previous research has linked sex steroids to nonverbal abilities [22–24]. In particular, androgens are hypothesized to have masculinizing effects on the fetal brain [15, 25]. Androgens may therefore contribute to improved performance in tasks that are typically associated with male superiority, such as nonverbal tasks. For example, females with congenital adrenal hyperplasia (a condition associated with high prenatal androgen exposure) have been shown to exhibit superior performance on tasks involving mental rotation [26–29]. In contrast, males with CAH show impaired performance relative to controls [30]. Furthermore, direct measurement in-utero of amniotic fluid testosterone has linked higher testosterone concentrations to a faster rate of rotation on a mental rotation task in girls, but a slower rate in boys [31]. Though the relationship between nonverbal abilities and sex steroids are not widely studied, a positive linear relationship has been found linking testosterone to nonverbal ability in males [32].

Whilst androgens have received the most attention in the literature, estrogens such as estradiol may promote brain masculinisation and/or defeminisation via aromatization (the conversion of androgens to estrogen), particularly in male rodents [33]. The relationship in humans between estrogens and nonverbal abilities is not as commonly examined. Sprague Dawley rats exposed to perinatal estradiol demonstrated improved accuracy in spatial ability on a radial-arm maze task [34]. Furthermore, levels of circulating estradiol have been linked to improved mental rotation task performance in women [35].

Prenatal sex steroids have less commonly been examined in relation to verbal abilities and findings are mixed. One study demonstrated an inverted u-shaped relationship between amniotic testosterone concentration and language comprehension and classification in 4-year-old girls, but not in boys [36]. Another study found that amniotic testosterone concentration was inversely related to vocabulary size at 18 and 24 months. However, this relationship was significant only when both sexes were examined together and not separately [37]. More recent studies involving the Western Australian Pregnancy (Raine) Cohort have demonstrated inverse relationships between perinatal bioavailable testosterone concentrations in umbilical cord blood and pragmatic language at age 10 in girls [38] and expressive vocabulary in boys at age 2 [39]. Higher cord-blood testosterone concentration has also been linked to significant language delay in males between the ages of 1 and 3 [40]. Interestingly, in this last study, perinatal testosterone was a protective factor in females between the ages of 1 and 3 whereby it was associated with improved language.

Overall, the existing research examining verbal and nonverbal abilities and sex steroids is inconsistent. There is significant variation in age of participants, sample size, sex steroid sampling method, and type of verbal and nonverbal abilities measured. The current study builds on previous literature in several ways. First, we examined a range of biologically active androgens including Androstenedione (A4) and dehydroepiandrosterone (DHEA), which have been associated with weak masculinizing effects [41, 42]. Moreover, these pro-hormones are also involved in sex steroid pathways that are closely linked to the synthesis of other androgens as well as estrogens [43]. We also investigated a range of biologically active estrogens (estrone, estetrol, estriol and estradiol). This enabled us to examine biologically active estrogens and androgens in relation to cognition.

Secondly, we examined sex steroids in a large pregnancy cohort by directly measuring sex steroids in umbilical cord blood at birth. Direct methods of sampling steroids include amniotic fluid, maternal blood and umbilical cord blood sampling. Whilst these methods all have strengths and weaknesses (see [44–46]), cord blood sampling is considered to be the most representative method whereby its comparatively less intrusive nature allows for the measurement of sex steroids in larger samples involving the general population.

Finally, no study has yet investigated sex steroids in cord blood and examined their relationship to both nonverbal and verbal cognitive abilities in middle childhood. Hence the current study attempted to do this. Androgen and estrogen composites were calculated according to a novel method proposed by Hollier et al. [47]. This method weights the biological activity of each steroid to form composites for both androgens and estrogens. These composites were then analysed in relation to verbal measures and nonverbal measures administered at age 10. Based on the existing literature it would be expected that females outperform males on verbal measures (CELF-3 and PPVT-III) and vice versa on the nonverbal measure (RCPM). In particular the RCPM has been found in a meta-analysis to demonstrate a small but significant advantage for males compared to females. The authors hypothesize that this may be due to the greater visualization component involved as compared to the original Standard Progressive Matrices in which sex differences are not apparent at age 10 [48]. In addition, the Androgen composite would be expected to negatively predict verbal scores and positively predict nonverbal scores and these effects may differ according to sex. The nature of the relationship between the Estrogen composite values and subsequent scores on verbal and nonverbal measures is unclear.

## Methods

### Participants

Participants were recruited from the Raine study in Western Australia. The original samples involved 2,900 women recruited from the public antenatal clinic at King Edward Memorial Hospital or surrounding private clinics between May 1989 and November 1991. The pregnant women had a gestational age of between 16 and 20 weeks, delivered at King Edward Memorial Hospital, had English language skills and had the intention to remain in Western Australia for subsequent follow ups [49]. In total, 2,868 (96%) births were available for follow-up. Written informed consent was obtained from mothers and guardians who participated, this was documented on paper and stored securely in a locked cabinet. The mothers provided consent when pregnant, parents or guardians at age 10. Participant recruitment, consent and all follow-ups were approved by the Human Ethics Committee at King Edward Memorial Hospital and/ or Princess Margaret Hospital for Children in Perth.

### Procedure

Cord blood was obtained from offspring of the Raine cohort at birth. The children were seen at multiple time points (every 2–3 years) for comprehensive phenotyping. At age 10, participants completed the Clinical Evaluation of Language Fundamentals-3 (CELF-3) and the Peabody Picture Vocabulary Test third edition (PPVT-III) as verbal measures and the Raven’s Coloured Progressive Matrices (RCPM) as a nonverbal measure.

### Measures

#### Sex steroid measurement

Mixed arterial and venous umbilical cord blood was collected at the birth of 860 deliveries as previously described [21]. Immediately after delivery, mixed umbilical arterial-venous (UA:UV) cord blood was collected, allowed to clot and the resulting serum was frozen at -80°C and stored without thawing until the present study was performed. Eight hundred and three cord blood samples (92.3%) representing 396 female and 407 male infants had sufficient serum (after removal, aliquoting and archiving of 1 ml for future studies) for steroid analysis. In January 2010, these serum samples were thawed and analysed for sex steroid content by liquid chromatography-tandem mass spectrometry described by Keelan et al. [50]. SHBG was measured by ELISA according to the manufacturer’s instructions (IBL International, Hamburg, Germany). The inter-assay imprecision was <4.5% (n = 25). Intra-assay variation was 5.2% (n = 860). Samples with an initial replicate coefficient of variation (CV) of >10% were reanalyzed [50]. The androgens measured were testosterone, androstenedione (A4) and dehydroepiandrosterone (DHEA). The estrogens examined were estrone (E1), estradiol (E2), estriol (E3) and estetrol (E4).

#### Calculation of bioavailable testosterone, estradiol and estrone

The following formula was used to calculate BioT (nmol/L), representing the fraction of total testosterone either free (unsequestered by SHBG) or bound to serum albumin [50].

BioT = [free testosterone] + [albumin−bound testosterone]

Free testosterone was calculated using the empirical method and formula described by [51]. Albumin levels were adjusted using published reference values to take into account the decrease in serum albumin concentrations with gestational age [52]. Bioavailable concentrations of E_1_ (BioE_1_) and of E_2_ (BioE_2_) were calculated using the method described by Mazer [53] and adjusted accordingly as described in detail by Hollier et al. (2014).

#### Composite measures

The calculated composites take into account the biological potency, binding affinity and unbound proportion of the sex steroids. Each steroid is weighted according to its biological potency and the following formulae are used:

$$\begin{array}{lll} Androgen\;Composite & = & BioT\;+\;0.1\left[A4\right]\;+\;0.01\left[DHEA\right]\\ Estrogen\;Composite & = & BioE2\;+\;0.5\left[BioE1\right]\;+\;0.1\left[E3\right]\;+\;0.02\left[E4\right] \end{array}$$

The formulae used to calculate the composites are explained in detail in Hollier et al. (2014).

#### The Clinical Evaluation of Language Fundamentals-3

The Clinical Evaluation of Language Fundamentals-3 (CELF-3; [54]) is a widely used language assessment that measures expressive and receptive language ability in individuals aged 6 to 21. The assessment takes approximately 30 to 45 minutes to complete. In children older than 9, the test includes two domains, with four subscales measuring receptive language (Concepts and Directions, Semantic Relations, Word Classes, Recalling Sentences) and three subscales measuring expressive language (Sentence Assembly, Formulated Sentences, Recalling Sentences). The CELF-3 has been shown to have sound psychometric properties including high internal consistency (.83 - .95). As part of the analyses of the current study, sex differences in CELF-3 scores are examined. For this reason we elected to use raw scores as opposed to the sex-specific standard scores.

#### The Peabody Picture Vocabulary Test third edition

The Peabody Picture Vocabulary Test third edition (PPVT-III;[55]) is a commonly administered standardised test examining verbal ability including receptive vocabulary knowledge and comprehension. It is an individually administered, norm referenced test designed for individuals aged 2.5 to 90. There are 204 items and each page consists of four black and white line drawings. The examiner instructs the participant to “Put your finger on ___, Show me ____, or Find” the named target stimulus. A raw score is calculated by summing scores across items and then a standard score (*M* = 100, *SD* = 15) is derived. The PPVT-III has sound reliability and validity [56, 57].

#### Raven’s Coloured Progressive Matrices

Raven’s Coloured Progressive Matrices (RCPM; [58]) is a widely used measure of nonverbal intelligence for individuals between the ages of 5 and 11. The test consists of 36-items that increase in difficulty. Each item consists of a matrix of geometric figures with one figure missing. Individuals then select an item from options below that best fits the visual pattern of the matrix. The psychometric properties of this measure in children are considered generally sound [59].

### Sample characteristics

Sociodemographic variables (maternal age at conception, maternal education, family income) were recorded at 18 weeks’ pregnancy, antenatal variables (maternal smoking and alcohol consumption during pregnancy) at 34 weeks’ pregnancy and obstetric variables (gestational age, offspring gender, parity, and Apgar scores) at birth. Proportion of optimal birth weight (POBW) was also calculated to provide an indicator of appropriateness of fetal growth [60]. This was based on the ratio of the observed birth weight to the optimal birth weight for that individual [61].

### Statistical analyses

Scores on the CELF-3 and PPVT-III were combined to form a composite verbal score. This composite score along with scores on RCPM were transformed to z-scores to provide standardized verbal and nonverbal ability scores. Those who completed measures at age 10 and those who did not were compared on sex steroid composites, and on obstetric, antenatal and sociodemographic variables. Independent-samples t-tests were conducted to compare the sexes on key sex steroid and verbal and nonverbal measures. Any subsequent analyses were sex specific. Correlation analyses were conducted to examine relationships between sex steroid and verbal and nonverbal measures in each sex. Spearman’s rho was used to examine monotonic relationships and curve estimation regression analyses were conducted in order to account for any quadratic relationships. Any significant correlations or regression models were followed up with a stepwise multiple regression to determine the predictive ability of the sex steroid composite in relation to the outcome variables. Any antenatal, obstetric or sociodemographic variable that significantly correlated (p <.05) with the verbal or non-verbal measure was entered in the first block. The composite was entered in the second block and any quadratic relationships were also investigated by entering the quadratic term of the sex steroid composite in the third block of the regression analysis. An alpha level of .05 was used for all statistical analyses.

## Results

There were 860 children who had available sex steroid data of which 464 (227 females and 237 males) provided follow-up information at age 10 years on the PPVT-III, RCPM and the CELF-3. There was no significant difference in age between males (*M* = 10.63, *SD* = 0.17) and females (*M* = 10.61, *SD* = .18) at the 10 year follow up, *t*(462) = .67, *p* = .*50*. Chi-squared analyses were conducted to examine any differences between those who did and did not complete the measures at age 10 –these are presented in Table 1. Those individuals who did not complete the measures at age 10 were more likely to have younger mothers, mothers who smoked during pregnancy and have a family income below the poverty line. In contrast, there was no significant difference found in maternal education at pregnancy, maternal alcohol intake during pregnancy, gestational age, POBW, sex, 5-minute Apgar score or parity. There were also no significant differences between completers and non-completers for females on the Androgen, *t*(426) = 1.14, *p* = .26, or Estrogen, *t*(426) = 0.42, *p* = .67, composite, and also for males on the Androgen, *t*(429) = 1.03, *p* = .31, or Estrogen, *t*(429) = .66, *p* = .51, composite.

**Table 1:** Characteristics of those who did and did not complete age 10 measures.

Categorical variables	Completed age 10 measures (n = 464)		Did not complete age 10 measures (n = 396)		*p* value
	N	n (%)	N	n (%)	
Maternal age at birth	453		376		<.01
<20		31 (6.8)		47 (12.5)	
20–24		84 (18.5)		93 (24.7)	
25–29		124 (27.4)		111 (29.5)	
30–34		140 (30.9)		84 (22.3)	
35+		74 (16.3)		41 (10.9)	
Maternal education at pregnancy	453		379		.51
Completed secondary school		170 (37.5)		134 (35.4)	
Did not complete secondary school		283 (62.5)		245 (64.6)	
Family income below poverty line	452		369		<.01
Yes		167 (36.9)		181 (49.1)	
No		285 (63.1)		188 (50.9)	
Maternal smoking in pregnancy	454		372		<.05
None		352 (77.5)		262 (70.4)	
1–10 cigarettes daily		62 (13.7)		55 (14.8)	
11+ cigarettes daily		40 (8.8)		55 (14.8)	
Maternal alcohol intake during preganancy	454		372		.30
None		273 (60.1)		242 (65.1)	
Once a week or less		156 (34.4)		109 (29.3)	
Several times a week or more		25 (5.5)		21 (5.6)	
Gestational age	453		376		.38
< 32 weeks		8 (1.8)		9 (2.4)	
32 to 37 weeks		72 (15.9)		68 (18.1)	
38 to 40 weeks		291 (64.2)		246 (65.4)	
> 40 weeks		82 (18.1)		53 (14.1)	
Proportion of optimal birthweight	462		395		.45
< 90		133 (28.8)		127 (32.2)	
90 to 110		263 (56.9)		208 (52.7)	
> 110		66 (14.3)		60 (15.2)	
Sex	464		395		.52
Male		233 (50.2)		198.2 (50.1)	
Female		231(49.8)		197 (49.9)	
Apgar score	453		375		.10
Generally normal		441 (97.4)		357 (95.2)	
Fairly low		12 (2.6)		18 (4.8)	
Critically low		0		0	
Parity	464		394		.06
0		203 (43.8)		198 (50.3)	
1		154 (33.2)		103 (26.1)	
>1		107 (23.1)		93 (23.6)	

### Sex differences on sex steroids and verbal and non-verbal measures

Table 2 presents the outcome of independent-samples t-tests conducted to examine sex differences on sex steroid composites and verbal and nonverbal measures and subscales. Males had a significantly higher Androgen composite than females, while there was no sex difference in the estrogen composite. Females scored significantly higher than males on the Word Classes and Sentence Assembly subscales of the CELF-3 (all *p*s *<*.05). No other measures exhibited a significant sex difference (all *p*s >.05).

**Table 2:** Mean (SD) for sex steroid variables and verbal and non-verbal measures at age 10 for males and females.

	Females (N = 231)	Males (N = 233)	*p* value	Cohen’s *d*
Androgen composite	0.38 (.16)	0.43 (.16)	.001**	0.31
Estrogen composite	94.73 (46.83)	97.68 (47.64)	.50	0.06
CELF-3 total score	97.31 (14.81)	94.75 (15.15)	.07	0.17
• Concepts and directions	24.53 (4.15)	24.24 (4.31)	.45	0.07
• Word classes	23.46 (4.57)	22.33 (5.41)	.02*	0.23
• Semantic relations	15.30 (4.17)	14.81 (4.59)	.23	0.11
• Sentence assembly	11.55 (4.20)	10.43 (4.63)	.01*	0.25
• Formulated sentences	29.43 (6.61)	28.82 (6.81)	.33	0.09
• Recalling sentences	47.47 (12.57)	46.32 (12.62)	.34	0.09
PPVT-III total standard score	103.17 (12.36)	103.65 (12.38)	.68	0.04
RCPM score	31.19 (3.57)	31.17 (3.35)	.96	0.01
Total verbal z-score	0.08 (1.82)	-0.052 (1.81)	.45	0.07

*Note*: PPVT-III = Peabody Picture Vocabulary Test—III, CELF-3 = Clinical Evaluation of Language Fundamentals -3, RCPM = Raven’s Coloured Progressive Matrices.

*p <.05,

**p< .01

### Relationships between sex steroid composite values and verbal and nonverbal measures

Table 3 shows the outcome of Spearman rank correlations conducted on sex steroid composites and nonverbal and verbal measures separately for each sex. In females, Androgen composite values were weakly negatively correlated with Total verbal standard scores and with the Concepts and Directions subscale scores of the CELF-3 (all *ps <* .*05*). For males, Androgen composite values were weakly negatively correlated with the Formulated Sentences subscale scores. All other correlations in males and females were non-significant (all *p*s >.05). Sex specific curve estimation regression analyses were carried out on all main outcome variables with no statistically significant effects (all ps > .05).

**Table 3:** Spearman’s correlations (p value) between sex steroid values and scores on measures for males and females.

	Androgen composite		Estrogen composite	
	Males	Females	Males	Females
CELF-3 total score	-.11 (.12)	-.13 (.05)	-.01 (.89)	.03 (.71)
• Concepts and directions	-.07 (.29)	-.16 (0.01)*	-.02 (.81)	-.01 (.86)
• Word classes	-.09 (.17)	-.09 (.20)	.06 (.37)	.01 (.84)
• Semantic relations	-.04 (.55)	-.10 (.13)	-.02 (.79)	.01 (.88)
• Sentence assembly	-.09 (.19)	-.10 (.15)	.03 (.69)	-.02 (.82)
• Formulated sentences	-.15 (.02)*	- .06 (.36)	-.01 (.94)	-.02 (.82)
• Recalling sentences	-.06 (.36)	-.10 (.15)	.08 (.22)	.05 (.47)
PPVT-III total standard score	-.10 (.12)	-.12 (.08)	-.09 (.90)	.01 (.87)
RCPM score	-.002 (.98)	.01 (.85)	-.06 (.40)	.06 (.33)
Total verbal z-score	-.11 (.10)	-.14 (.04)*	.04 (.51)	.01 (.84)

*Note*: PPVT-III = Peabody Picture Vocabulary Test—III, CELF-3 = Clinical Evaluation of Language Fundamentals -3, RCPM = Raven’s Coloured Progressive Matrices.

*p <.05

Further hierarchical multiple linear regression analyses were conducted as a follow-up to the significant correlations. Covariates that significantly correlated with the outcome variable were included in the first step, the Androgen composite at the second step, and the quadratic Androgen composite at the third step of the model to account for possible non-linear effects. In females, Androgen composite values did not significantly predict total Verbal scores (Table 4) or scores on the Concepts and Directions subscale (see Table 5) over and above the variance accounted for by covariates. In males, the quadratic Androgen composite did significantly predict scores on the Formulated Sentences subscale over and above the variance accounted for by covariates (see Table 6).

**Table 4:** Outcomes of hierarchical multiple regression analyses predicting the total verbal scores in females.

		*B*	*SE B*	*B*	R^2^	R^2^ change
Step 1	Covariates only				.08	
Step 2	Constant	.66	.33			
	Maternal education	.65	.26	.17		
	Maternal income	-.76	.26	-.20		
	Androgen composite	-1.35	.74	-.12		
					.10	.01, *p* = .07
Step 3	Constant	1.22	.51			
	Maternal education	.68	.26	.18		
	Maternal income	-.73	.26	-.19		
	Androgen composite	-4.27	2.17	-38		
	Quadratic androgen composite	3.10	2.16	.27		
					.10	.01, *p* = .15

**Table 5:** Outcomes of hierarchical multiple regression analyses predicting the concepts and directions subscale score in females.

		*B*	*SE B*	*B*	R^2^	R^2^ change
Step 1	Covariates only				.09	
Step 2	Constant	23.44	1.40			
	Maternal education	1.24	.58	.15		
	Maternal income	-1.40	.57	-.16		
	Maternal language	.70	1.17	.04		
	Maternal race	1.86	.94	.14		
	Androgen composite	-2.96	1.23	-.16		
					.11	.01, *p* = .08
Step 3	Constant	24.01	1.62			
	Maternal education	1.27	.59	.15		
	Maternal income	-1.37	.58	-.16		
	Maternal language	.75	1.17	.05		
	Maternal race	1.86	.94	.14		
	Androgen composite	-6.14	4.84	-.24		
	Quadratic androgen composite	3.45	4.85	.14		
					.11	.002, *p* = .48

**Table 6:** Outcomes of hierarchical multiple regression analyses predicting the formulated sentences subscale score in males

		*B*	*SE B*	*B*	R^2^	R^2^ change
Step 1	Covariates only				.13	
Step 2	Constant	25.43	2.65			
	Maternal education	1.57	.95	.11		
	Maternal income	-3.57	.96	-.25		
	Maternal race	2.97	1.67	.12		
	Birth weight category	.92	.70	.09		
	Maternal alcohol consumption	1.24	.75	.11		
	Androgen composite	-1.82	2.83	-.04		
					.13	.002, *p* = .52
Step 3	ConstantQuadratic androgen composite	29.8519.59	3.298.77	.49		
	Maternal education	1.61	.94	.11		
	Maternal income	-3.32	.96	-.23		
	Maternal race	3.11	1.66	.12		
	Birth weight category	.95	.69	.09		
	Maternal alcohol consumption	1.31	.74	.11		
	Androgen composite	-22.36	9.61	-.51		
					.15	.02, *p* = .03

## Discussion

The current study examined the relationship between sex steroids in umbilical cord blood and nonverbal and verbal abilities in middle childhood. As expected, androgen composite values were significantly higher in males than females. This supports commonly acknowledged sex differences in testosterone concentrations found prenatally and at birth, in both animal and human studies [62–64]. However, the Estrogen composite was not found to differ according to sex. This too is unsurprising given the limited effects found so far in the existing literature when examining estrogens both prenatally and perinatally [65, 66].

Raw scores on the Word Classes and Sentence Assembly subscales of the CELF-3 were significantly higher for females than males, though this effect was small in magnitude. Whilst the CELF-3 subscale scores appear to trend towards a slight female advantage, statistically the majority of the verbal measures do not support a significant sex difference in performance. Age factors may explain, in part, the lack of sex differences in these results. For example, the variation between sexes in language ability appears to be most prominent at early preschool ages. A meta-analysis found that verbal superiority in females was apparent in those younger than age 5 and older than 26 [10]. It may be that age effects interact with the type of verbal ability being assessed. For example, sex differences appear to be more pronounced in early vocabulary development [39, 67, 68]. Overall, these results support recent suggestions of the inconsistency of female superiority on language measures [13, 69].

The lack of sex differences found on the RCPM is inconsistent with the common perception of superior nonverbal ability in males. Research on sex differences in this area tap into multiple components of nonverbal ability and tend to show the most pronounced effects for mental rotation tasks [5]. Whilst previous meta-analyses found a small effect of male superiority in children aged 5–11 on the RCPM [48]. This may be attributed to age specific effects and ongoing maturation of the brain. For example, much of the prefrontal cortex development is thought to occur in middle childhood [70].

Higher Androgen composites were related to lower scores on the Concepts and Directions subscale and Total verbal z-score in girls. However, these relationships did not remain significant when covariates were taken into account. Language development does not occur solely in response to genetic and biological influences. Whilst the current study attempted to control for possible environmental covariates such as socioeconomic status, maternal language and race, language development occurs in response to complex environmental factors which influence subsequent performance [71–73]. Higher Androgen Composite values did demonstrate a u-shaped relationship to Formulated Sentences subscale scores in males suggesting that the ability to produce grammatically correct sentences is highest in individuals with either high or low concentrations of androgens. However this effect was small as quadratic Androgen composite values accounted for approximately 2% of additional variance. There were no relationships found between estrogen or androgen composite values and the PPVT-III or RCPM scores. Overall, these results suggest that at age 10, perinatal androgen and estrogen were not related in any substantial way to verbal or nonverbal measures.

There are a number of alternative explanations for the null results found in the current study. First, few of the outcome variables demonstrated sex differences. Prenatal sex steroids are more likely to be related to outcome measures that demonstrate clear differences between sexes [18]. The verbal and spatial measures used in the current study may therefore tap into areas in which cognitive sex differentiation is less pronounced and where the differentiation may be age or task-related, as previously discussed. It should also be noted that verbal and nonverbal abilities are umbrella terms that encompass a range of sub-constructs that are, at present, still unclearly differentiated [74]. The current study is therefore only indicative of the specific abilities which these tasks measure.

Future research could attempt to compartmentalize these areas further in order to untangle the areas of cognition in which sex steroids may play a part. This study therefore does not undermine the previous findings in cord blood linking prenatal testosterone to early language development [38, 39]. It should be noted that there is merit in continuing to use less invasive, more representative methods such as cord blood sampling and the examination of a wide scope of sex steroids, particularly using reliable methods such as mass spectrometry [50]. Further research of this kind would allow us to more accurately define the parameters under which these late gestation levels of hormones may influence certain aspects of cognition, such as verbal and nonverbal abilities. This research is informative in exploring the traditional male-female divide in verbal and nonverbal abilities and the possible mechanisms influencing early neurocognitive development.

## References

[1] Halpern DF. Sex Differences in Cognitive Abilities: 4th Edition: Taylor & Francis; 2013.

[2] Fairweather H. Sex differences in cognition. Cognition. 1976;4(3):231–80.

[3] Waber DP. Sex differences in cognition: A function of maturation rate? Science. 1976;192(4239):572–4. 1257795 10.1126/science.1257795

[4] Kimura D. Sex Differences in Cognition. Encyclopedia of Cognitive Science. 2003.

[5] Voyer D, Voyer S, Bryden MP. Magnitude of sex differences in spatial abilities: a meta-analysis and consideration of critical variables. Psychological bulletin. 1995;117(2):250 7724690 10.1037/0033-2909.117.2.250

[6] Moore DS, Johnson SP. Mental rotation in human infants a sex difference. Psychological Science. 2008;19(11):1063–6. 10.1111/j.1467-9280.2008.02200.x 19076473 PMC2651884

[7] Quinn PC, Liben LS. A sex difference in mental rotation in young infants. Psychological Science. 2008;19(11):1067–70. 10.1111/j.1467-9280.2008.02201.x 19076474

[8] Linn MC, Petersen AC. Emergence and characterization of sex differences in spatial ability: A meta-analysis. Child development. 1985:1479–98. 4075870

[9] Voyer D. Time limits and gender differences on paper-and-pencil tests of mental rotation: a meta-analysis. Psychonomic bulletin & review. 2011;18(2):267–77. 21327340 10.3758/s13423-010-0042-0

[10] Hyde JS, Linn MC. Gender differences in verbal ability: A meta-analysis. Psychological bulletin. 1988;104(1):53.

[11] Hedges LV, Nowell A. Sex differences in mental test scores, variability, and numbers of high-scoring individuals. Science. 1995;269(5220):41–5. 7604277 10.1126/science.7604277

[12] Hines M. Sex-related variation in human behavior and the brain. Trends in cognitive sciences. 2010;14(10):448–56. 10.1016/j.tics.2010.07.005 20724210 PMC2951011

[13] Hyde JS. Gender similarities and differences. Annual review of psychology. 2014;65:373–98. 10.1146/annurev-psych-010213-115057 23808917

[14] Hines M. Gender development and the human brain. Annual review of neuroscience. 2011;34:69–88. 10.1146/annurev-neuro-061010-113654 21438685

[15] Phoenix CH, Goy RW, Gerall AA, Young WC. Organizing action of prenatally administered testosterone propionate on the tissues mediating mating behavior in the female guinea pig. Endocrinology. 1959;65(3):369–82. 14432658 10.1210/endo-65-3-369

[16] Valla JM, Ceci SJ. Can sex differences in science be tied to the long reach of prenatal hormones? Brain organization theory, digit ratio (2D/4D), and sex differences in preferences and cognition. Perspectives on Psychological Science. 2011;6(2):134–46. 22164187 10.1177/174569161140023 PMC3230041

[17] Jacklin CN, Wilcox KT, Maccoby EE. Neonatal sex-steroid hormones and cognitive abilities at six years. Developmental Psychobiology. 1988;21(6):567–74. 10.1002/dev.420210607 3169381

[18] Auyeung B, Lombardo MV, Baron-Cohen S. Prenatal and postnatal hormone effects on the human brain and cognition. Pflügers Archiv-European Journal of Physiology. 2013;465(5):557–71. 10.1007/s00424-013-1268-2 23588379

[19] Auyeung B, Baron-Cohen S. Fetal testosterone in mind: Human sex differences and autism. The Primate Mind: Built to Connect with Other Minds. 2012:194.

[20] Baron-Cohen S, Knickmeyer RC, Belmonte MK. Sex differences in the brain: implications for explaining autism. Science. 2005;310(5749):819–23. 10.1126/science.1115455 16272115

[21] Jamnadass ESL, Keelan JA, Hollier LP, Hickey M, Maybery MT, Whitehouse AJO. The perinatal androgen to estrogen ratio and autistic-like traits in the general population: a longitudinal pregnancy cohort study. Journal of Neurodevelopmental Disorders. 2015;7(1):17 10.1186/s11689-015-9114-9 26085846 PMC4470005

[22] Hampson E. Spatial cognition in humans: possible modulation by androgens and estrogens. Journal of Psychiatry and Neuroscience. 1995;20(5):397 8527425 PMC1188722

[23] Auyeung B, Knickmeyer R, Ashwin E, Taylor K, Hackett G, Baron-Cohen S. Effects of fetal testosterone on visuospatial ability. Archives of sexual behavior. 2012;41(3):571–81. 10.1007/s10508-011-9864-8 22033667

[24] Tan Ü. Relationship of testosterone and nonverbal intelligence to hand preference and hand skill in right-handed young adults. International Journal of Neuroscience. 1990;54(3–4):283–90. 2265977 10.3109/00207459008986645

[25] Hines M. Brain gender: Oxford University Press; 2003.

[26] Resnick SM, Berenbaum SA, Gottesman II, Bouchard TJ. Early hormonal influences on cognitive functioning in congenital adrenal hyperplasia. Developmental Psychology. 1986;22(2):191.

[27] Hampson E, Rovet JF, Altmann D. Spatial reasoning in children with congenital adrenal hyperplasia due to 21-hydroxylase deficiency. Developmental Neuropsychology. 1998;14(2–3):299–320.

[28] Hampson E, Rovet JF. Spatial function in adolescents and young adults with congenital adrenal hyperplasia: Clinical phenotype and implications for the androgen hypothesis. Psychoneuroendocrinology. 2015;54:60–70. 10.1016/j.psyneuen.2015.01.022 25686803

[29] Berenbaum SA, Bryk KLK, Beltz AM. Early androgen effects on spatial and mechanical abilities: evidence from congenital adrenal hyperplasia. Behavioral neuroscience. 2012;126(1):86 10.1037/a0026652 22289044 PMC3270350

[30] Puts DA, McDaniel MA, Jordan CL, Breedlove SM. Spatial ability and prenatal androgens: meta-analyses of congenital adrenal hyperplasia and digit ratio (2D: 4D) studies. Archives of sexual behavior. 2008;37(1):100–11. 10.1007/s10508-007-9271-3 18074217 PMC2883918

[31] Grimshaw GM, Sitarenios G, Finegan J-AK. Mental rotation At 7 years-relations with prenatal testosterone levels and spatial play experiences. Brain and cognition. 1995;29(1):85–100. 10.1006/brcg.1995.1269 8845125

[32] Tan Ü, Akgün A. There is a direct relationship between nonverbal intelligence and serum testosterone level in young men. International journal of neuroscience. 1992;64(1–4):213–6. 1342041 10.3109/00207459209000548

[33] Wallen K, Baum MJ. Masculinization and defeminization in altricial and precocial mammals: comparative aspects of steroid hormone action. Hormones, brain and behavior. 2002;4:385–423.

[34] Williams CL, Meck WH. The organizational effects of gonadal steroids on sexually dimorphic spatial ability. Psychoneuroendocrinology. 1991;16(1):155–76. 1961837 10.1016/0306-4530(91)90076-6

[35] Hampson E, Levy-Cooperman Na, Korman JM. Estradiol and mental rotation: relation to dimensionality, difficulty, or angular disparity? Hormones and behavior. 2014;65(3):238–48. 10.1016/j.yhbeh.2013.12.016 24394702

[36] Finegan J-AK, Niccols GA, Sitarenios G. Relations between prenatal testosterone levels and cognitive abilities at 4 years. Developmental Psychology. 1992;28(6):1075.

[37] Lutchmaya S, Baron-Cohen S. Human sex differences in social and non-social looking preferences, at 12 months of age. Infant Behavior and Development. 2002;25(3):319–25.

[38] Whitehouse AJ, Maybery MT, Hart R, Mattes E, Newnham JP, Sloboda DM, et al Fetal androgen exposure and pragmatic language ability of girls in middle childhood: implications for the extreme male-brain theory of autism. Psychoneuroendocrinology. 2010;35(8):1259–64. 10.1016/j.psyneuen.2010.02.007 20206450

[39] Hollier LP, Mattes E, Maybery MT, Keelan JA, Hickey M, Whitehouse AJ. The association between perinatal testosterone concentration and early vocabulary development: A prospective cohort study. Biological psychology. 2013;92(2):212–5. 10.1016/j.biopsycho.2012.10.016 23153707

[40] Whitehouse AJ, Mattes E, Maybery MT, Sawyer MG, Jacoby P, Keelan JA, et al Sex-specific associations between umbilical cord blood testosterone levels and language delay in early childhood. Journal of Child Psychology and Psychiatry. 2012;53(7):726–34. 10.1111/j.1469-7610.2011.02523.x 22276678

[41] Kraus C, Pfannkuche K, Trillmich F, Groothuis TG. High maternal androstenedione levels during pregnancy in a small precocial mammal with female genital masculinisation. Max Planck Institute for Demographic Research Working Papers. 2008;1:2008.

[42] Hutson JM. The hormones regulating sex development Disorders of Sex Development: Springer; 2012 p. 23–9.

[43] Dart D. Androgens have forgotten and emerging roles outside of their reproductive functions, with implications for diseases and disorders. J Endocr Disord. 2014;1(1):1005.

[44] Cohen-Bendahan CCC, van de Beek C, Berenbaum SA. Prenatal sex hormone effects on child and adult sex-typed behavior: methods and findings. Neuroscience & Biobehavioral Reviews. 2005;29(2):353–84. 15811504 10.1016/j.neubiorev.2004.11.004

[45] Hollier LP, Keelan JA, Hickey M, Maybery MT, Whitehouse AJ. Measurement of androgen and estrogen concentrations in cord blood: accuracy, biological interpretation, and applications to understanding human behavioral development. Frontiers in Endocrinology. 2014;5:64 10.3389/fendo.2014.00064 24829559 PMC4014673

[46] Knickmeyer R, Auyeung B, Davenport ML. Assessing prenatal and neonatal gonadal steroid exposure for studies of human development: methodological and theoretical challenges. Frontiers in endocrinology. 2014;5. 10.3389/fendo.2014.00242 PMC4294212 25642209

[47] Hollier LP, Keelan JA, Jamnadass SL, Maybery MT, Hickey M, Whitehouse AJO. Adult Digit Ratio (2D:4D) is Not Related to Umbilical Cord Androgen or Estrogen Concentrations Early Human Development. 2014. 10.1016/j.earlhumdev.2014.12.011 25594498

[48] Lynn R, Irwing P. Sex differences on the progressive matrices: A meta-analysis. Intelligence. 2004;32(5):481–98.

[49] Newnham JP, Evans SF, Michael CA, Stanley FJ, Landau LI. Effects of frequent ultrasound during pregnancy: a randomised controlled trial. The Lancet. 1993;342(8876):887–91. 10.1016/0140-6736(93)91944-h 8105165

[50] Keelan JA, Mattes E, Tan H, Dinan A, Newnham JP, Whitehouse AJO, et al Androgen Concentrations in Umbilical Cord Blood and Their Association with Maternal, Fetal and Obstetric Factors. PLoS ONE. 2012;7(8):e42827 10.1371/journal.pone.0042827 22916165 PMC3423422

[51] Sartorius G, Ly LP, Sikaris K, McLachlan R, Handelsman DJ. Predictive accuracy and sources of variability in calculated free testosterone estimates. Annals of Clinical Biochemistry. 2009;46(2):137–43. 19225026 10.1258/acb.2008.008171

[52] Zlotkin S, Casselman C. Percentile estimates of reference values for total protein and albumin in sera of premature infants (less than 37 weeks of gestation). Clinical Chemistry. 1987;33(3):411–3. 3102125

[53] Mazer NA. A novel spreadsheet method for calculating the free serum concentrations of testosterone, dihydrotestosterone, estradiol, estrone and cortisol: With illustrative examples from male and female populations. Steroids. 2009;74(6):512–9. 10.1016/j.steroids.2009.01.008. 19321131

[54] Semel E, Wiig E, Secord W. CELF-3. Clinical evaluation of language fundamentals Spanish Edition San Antonio TX: The Psychological Corporation 1997.

[55] Dunn LM, Dunn LM. Peabody Picture Vocabulary Test: PPVT-IIIB: American Guidance Service Circle Pines, MN; 1997.

[56] Bochner S. Reliability of the Peabody Picture Vocabulary Test: A review of 32 selected research studies published between 1965 and 1974. Psychology in the Schools. 1978;15(3):320–7.

[57] Reynolds CR, Fletcher-Janzen E. Encyclopedia of Special Education: Wiley; 2007.

[58] Raven JC. Progressive matrices: A perceptual test of intelligence. London: HK Lewis; 1938.

[59] Raven J. Raven progressive matrices Handbook of nonverbal assessment: Springer; 2003 p. 223–37.

[60] Blair EM, Liu Y, de Klerk NH, Lawrence DM. Optimal fetal growth for the Caucasian singleton and assessment of appropriateness of fetal growth: an analysis of a total population perinatal database. BMC pediatrics. 2005;5(1):13 10.1186/1471-2431-5-13 15910694 PMC1174874

[61] Seron-Ferre M, Ducsay CA, Valenzuela GJ. Circadian Rhythms During Pregnancy. Endocrine Reviews. 1993;14(5):594–609. 10.1210/edrv-14-5-594 8262008

[62] Slob A, Ooms M, Vreeburg J. Prenatal and early postnatal sex differences in plasma and gonadal testosterone and plasma luteinizing hormone in female and male rats. Journal of Endocrinology. 1980;87(1):81–7. 7430918 10.1677/joe.0.0870081

[63] Abramovich D. Human sexual differentiation—in utero influences. BJOG: An International Journal of Obstetrics & Gynaecology. 1974;81(6):448–53. 10.1111/j.1471-0528.1974.tb00494.x 4407079

[64] Knickmeyer R, Baron-Cohen S. Fetal testosterone and sex differences. Early Human Development. 2006;82(12):755–60. 10.1016/j.earlhumdev.2006.09.014 17084045

[65] Lutchmaya S, Baron-Cohen S, Raggatt P, Knickmeyer R, Manning JT. 2nd to 4th digit ratios, fetal testosterone and estradiol. Early Human Development. 2004;77(1):23–8. 15113628 10.1016/j.earlhumdev.2003.12.002

[66] Resko JA, Ploem JG, Stadelman HL. Estrogens in fetal and maternal plasma of the rhesus monkey. Endocrinology. 1975;97(2):425–30. 10.1210/endo-97-2-425 808410

[67] Galsworthy MJ, Dionne G, Dale PS, Plomin R. Sex differences in early verbal and non-verbal cognitive development. Developmental Science. 2000;3(2):206–15.

[68] Huttenlocher J, Haight W, Bryk A, Seltzer M, Lyons T. Early vocabulary growth: Relation to language input and gender. Developmental psychology. 1991;27(2):236.

[69] Wallentin M. Putative sex differences in verbal abilities and language cortex: A critical review. Brain and language. 2009;108(3):175–83. 10.1016/j.bandl.2008.07.001 18722007

[70] Andersen SL. Trajectories of brain development: point of vulnerability or window of opportunity? Neuroscience & Biobehavioral Reviews. 2003;27(1):3–18. 12732219 10.1016/s0149-7634(03)00005-8

[71] Pungello EP, Iruka IU, Dotterer AM, Mills-Koonce R, Reznick JS. The effects of socioeconomic status, race, and parenting on language development in early childhood. Developmental psychology. 2009;45(2):544 10.1037/a0013917 19271838

[72] Alwin DF, McCammon RJ. Aging, cohorts, and verbal ability. The Journals of Gerontology Series B: Psychological Sciences and Social Sciences. 2001;56(3):S151–S61. 10.1093/geronb/56.3.s151 11316840

[73] Hoff-Ginsberg E. The relation of birth order and socioeconomic status to children's language experience and language development. Applied Psycholinguistics. 1998;19(04):603–29.

[74] Hegarty M, Waller D. Individual differences in spatial abilities The Cambridge handbook of visuospatial thinking. 2005:121–69.

